# Curae de Mim (Care for Me): A Personalized Alzheimer’s Care Nursing Intervention for Informal Caregivers

**DOI:** 10.3390/jpm15070270

**Published:** 2025-06-25

**Authors:** Catarina Inês Costa Afonso, Ana Spínola Madeira, Alcinda Reis, João Gomes

**Affiliations:** 1 Integrated Member of RISE-Health, School of Health Sciences of Santarém, Polytechnic Institute of Santarém, Quinta do Mergulhão Srª da Guia, 2005-075 Santarém, Portugal; ana.madeira@essaude.ipsantarem.pt (A.S.M.); alcinda.reis@essaude.ipsantarem.pt (A.R.); 2 Local Health Unit of the Leiria Region, 2400-441 Leiria, Portugal; joaomfg@gmail.com

**Keywords:** informal caregiver, Alzheimer’s disease, psychoeducational intervention, mental health, narrative care, personalized nursing, community health

## Abstract

**Background:** Informal caregivers of individuals with Alzheimer’s disease often experience high levels of emotional, physical, and psychological burden. Personalized nursing interventions are essential to support these caregivers and promote their well-being. **Objectives:** The objective of this study was to implement and evaluate a personalized psychoeducational intervention—Curae de Mim (Care for Me)—designed to reduce caregivers’ burden and enhance the emotional resilience among informal caregivers for people with Alzheimer’s disease. **Methods:** A mixed-methods study was conducted with 14 informal caregivers in a Portuguese community healthcare setting. The intervention consisted of six weekly group sessions guided by a mental health nurse, using cognitive–behavioral and recovery-oriented approaches. **Results:** After the intervention, the caregivers’ burden scores decreased significantly. The mean burden score dropped from 78 to 50. The thematic analysis revealed two key outcomes: emotional empowerment through peer interactions and reframing of the caregiver’s role through knowledge and self-care. **Conclusions:** This program proved effective in reducing caregivers’ burden and promoting adaptive coping. The integration of narrative reflection and specialized nursing care contributed to improved mental health outcomes.

## 1. Introduction

The increasing complexity of the mental health challenges associated with population aging has heightened the need for structured interventions that support both individuals living with dementia and their informal caregivers. In this context, informal caregivers refer to family members, friends, or other non-professional individuals who provide regular, unpaid assistance with daily living and emotional support to dependent persons, often in home settings. Globally, informal caregivers represent a critical—yet frequently undervalued—component of health systems, with approximately 80% of long-term care provided outside formal services [1,2].

Caring for a person with Alzheimer’s disease extends beyond physical support and often encompasses intensive emotional, psychological, and social challenges. Caregivers are at a high risk of developing depression, anxiety, and burnout, which in turn negatively impact their quality of life and personal health outcomes [3,4]. These effects are exacerbated when caregivers lack emotional resources, adequate training, and structured institutional support [5,6]. In Portugal, Sequeira and Sampaio [7] have emphasized that personalized nursing interventions are essential for improving caregivers’ coping mechanisms and reducing stress-related outcomes.

Several countries have developed national frameworks to support informal caregivers, recognizing their vital role in long-term dementia care. In Canada, the Caregiver Support Framework under the Canadian Dementia Strategy promotes the integration of community-based mental health resources, respite care, and caregiver education [8]. In the United Kingdom, the National Dementia Strategy encourages early intervention, individualized care plans, and structured psychoeducational programs [9]. Germany’s Care Time Act allows caregivers to take temporary paid leave, offering them financial protection and psychological relief [10]. Similarly, Australia’s Carer Gateway provides centralized access to emotional support services, skills training, and peer support [11].

These international experiences underline the importance of integrating psychosocial support into healthcare policy and primary care delivery to alleviate the caregiver burden and promote the sustainability of long-term care. In Portugal, although legislative advances such as the Statute of the Informal Caregiver and updates to the National Mental Health Program mark important progress, the operationalization of caregiver support remains fragmented and inconsistent [12].

Mental health and psychiatric nurses are key actors in addressing these gaps. Their competencies in community mental health, psychoeducation, and therapeutic engagement position them to lead evidence-based, person-centered interventions [13].

This project was guided by the Tidal Model [14], a recovery-oriented framework that emphasizes the co-construction of meaning through dialogue and narrative. By focusing on caregivers’ emotional regulation, self-awareness, and personal coping strategies, the intervention was aligned with both national priorities and the international best practices [14,15].

The program developed focuses on a personalized psychoeducational intervention designed specifically for carers of people with Alzheimer’s disease. It aims to alleviate emotional distress, foster resilience, and enhance effective caregiving practices while prioritizing caregivers’ mental health and well-being. By aligning local needs with global evidence, this initiative contributes to the development of interdisciplinary, community-based approaches that strengthen caregiver support and promote continuity of care. The objective of this study was to implement and evaluate a personalized psychoeducational intervention—Curae de Mim (Care for Me)—designed to reduce caregivers’ burden and enhance the emotional resilience among informal caregivers of people with Alzheimer’s disease.

## 2. Methods

### 2.1. The Study Design

This was an explanatory sequential mixed-methods study, combining quantitative and qualitative data within a participatory, practice-based research approach. Quantitative data were collected and analyzed first, followed by qualitative data, aiming to contextualize and deepen the understanding of the quantitative findings [16]. It was developed as part of a continuous quality improvement initiative in a primary healthcare setting—specifically, within a community healthcare unit—during a clinical internship integrated into the master’s degree in Mental Health and Psychiatric Nursing.

This study followed the logic of action-research, aiming to respond to a real and previously identified problem: the psychosocial vulnerability and burden experienced by informal caregivers of persons with dementia. It was conducted in accordance with the Declaration of Helsinki and approved by the Health Ethics Committee of the Regional Health Ethics Committee (protocol no. 25/2021; approval date: 6 May 2021).

### 2.2. The Participants and the Setting

The participants were informal caregivers of individuals diagnosed with Alzheimer’s disease or other forms of dementia, followed by the primary healthcare team. This was made up of general practitioners, family nurses, and community health professionals, as is typical in the Portuguese National Health Service. The inclusion criteria included (i) being the main caregiver; (ii) providing care for at least six months; and (iii) agreeing to participate in the intervention sessions. The exclusion criteria included cognitive impairments or active psychiatric disorders that would interfere with participation, as assessed by the referring health professional.

Participants were recruited by the mental health nurse through direct referral from the primary healthcare team. Eligible caregivers were personally contacted; informed about the objectives, structure, and voluntary nature of the study; and given an informed consent form to review and sign. This process adhered to ethical guidelines and was approved by the Regional Health Ethics Committee (protocol no. 25/2021; approval date: 6 May 2021).

The setting was a community care unit located within an urban primary healthcare cluster in central Portugal. The intervention was coordinated by a mental health and psychiatric nursing specialist, in collaboration with a multidisciplinary team.

### 2.3. The Intervention Program

The intervention program was implemented as the core component of the explanatory sequential mixed-methods study. The intervention program consisted of six structured group sessions (weekly, 60–90 min each) delivered over six weeks. The sessions were also delivered using psychoeducational and psychotherapeutic strategies based on recovery-oriented and person-centered approaches [17,18]. Each session was designed around specific themes:

1. Understanding dementia: identifying the caregiver’s role and responsibilities;
2. Emotional regulation strategies and stress management;
3. Self-care techniques and time management;
4. Enhancing caregiver–patient communication;
5. Coping mechanisms for emotional and psychological distress;
6. Evaluating progress and reinforcing learned strategies.

The sessions were guided by a trained mental health nurse and followed a cognitive–behavioral framework to promote adaptive coping strategies.

Each caregiver participated in one-on-one sessions with a mental health and psychiatric nurse specialist, who tailored the content, strategies, and pacing to the caregiver’s specific needs, emotional condition, and caregiving context. This individualized approach ensured that the intervention was not only therapeutically responsive but also aligned with the core principles of person-centered mental healthcare, based on each caregiver’s emotional profile, caregiving context, and reflective input, making the intervention both person-centered and therapeutically responsive.

The quantitative component of this study was embedded within this intervention: caregiver burden was assessed at two time points—before the first session (the baseline) and after the final session (post-intervention)—using the Caregiver Burden Scale, a validated Portuguese-language instrument.

### 2.4. Data Collection and Evaluation

Following the structure of an explanatory sequential design, the data collection was conducted in two phases:

(1) The Quantitative Phase (Pre–Post-Evaluation):

Quantitative data were obtained using the Caregiver Burden Scale (CBS) [19], which was administered at two time points—prior to the intervention and upon its completion—to assess the changes in perceived caregiver burden. Descriptive statistics, including frequencies and percentages, were used to characterize the categorical variables. Given the limited sample size (n = 14) and the non-normal distribution observed in several variables, measures of the central tendency and dispersion for continuous data were expressed using medians and standard deviations, rather than means and interquartile ranges. In the descriptive analysis of the sociodemographic data, the number (n), percentage (%), mean (M), standard deviation (SD), minimum (Min), and maximum (Max) were used. To evaluate the pre–post differences in the CBS scores, the Wilcoxon signed-rank test—a non-parametric statistical method suitable for small, paired samples—was employed. All statistical analyses were performed using IBM SPSS Statistics, version 29.0, with the two-tailed significance level set at α = 0.05.

(2) The Qualitative Phase (Narrative Exploration):

After the intervention, qualitative data were gathered to contextualize and deepen the understanding of the quantitative results. Two primary sources informed this phase: reflective narratives written by the caregivers at the end of each session, offering insights into their thoughts, emotions, and practical experiences during the intervention, and systematic field notes recorded systematically by the mental health nurse–researcher, capturing the group dynamics, expressions of emotional change, and non-verbal cues.

Qualitative data were analyzed through a thematic analysis, following Braun and Clarke’s six-phase method [20]. This allowed for the identification of patterns of meaning that explained the individual and group-level changes observed in the burden scores. Individual cases were not described in the quantitative analysis; however, selected personal reflections are presented in the qualitative results to illustrate experiential variation.

(3) Integration and Interpretation:

Data integration occurred during the interpretation phase, where the qualitative findings were used to explain the quantitative trends, thereby reinforcing the explanatory nature of the design. Triangulation of the data (scales, narratives, and field notes), peer debriefing, and reflexive journaling supported the methodological rigor and trustworthiness of the findings. This comprehensive approach ensured a robust and context-sensitive understanding of the caregivers’ experiences and the effectiveness of the intervention.

### 2.5. Ethical Considerations

This study was approved by the Health Ethics Committee of the Regional Health Ethics Committee (protocol code nº 25/2021; approval date: 6 May 2021) in accordance with the national and international guidelines for research in human subjects. All of the participants provided written informed consent after being duly informed about the objectives and procedures, the voluntary nature of participation, and their right to withdraw at any time. Anonymity and confidentiality were strictly maintained throughout the research process.

### 2.6. Rigor and Reliability

In mixed-methods research, particularly in the qualitative component, methodological rigor is ensured through strategies that strengthen the credibility, dependability, confirmability, and transferability of the findings. Unlike quantitative approaches, where the reliability is based on statistical consistency, qualitative rigor relies on the transparency, coherence, and traceability of the research process [16,21].

To ensure trustworthiness in this study, several methodological strategies were implemented: triangulation of the data sources, including questionnaires, narratives, and field notes; peer debriefing and validation of the interpretative findings within the research team [20]; an audit trail through systematic documentation of the decisions made during the data collection and analysis; reflexivity, supported by a reflexive journal maintained by the researcher, which helped to acknowledge and minimize potential biases; and verbatim transcription of the participants’ narratives to ensure fidelity to the original content.

These procedures contribute to the methodological robustness of this study and support the transferability of the insights to similar clinical settings, particularly within the scope of mental health nursing.

## 3. Results

A structured psychoeducational intervention was implemented in a community health setting over a period of 10 weeks. A total of 14 informal caregivers participated in this study and completed the full cycle of six sessions.

Table 1 shows the informal caregivers’ demographic characteristics. The caregivers were aged between 45 and 80 years, with a mean age of 61.5 years (SD = 11.72). On average, the participants had been in a caregiving role for 3.6 years, ranging from 1 to 11 years (SD = 3.52) (Table 1). The amount of time they dedicated to caregiving ranged from 5 to 24 h per day, with a mean of 18.21 h (SD = 6.7) and a mode of 24 h. Consistent with the existing literature, the majority of caregivers were women (78.3%), primarily spouses or daughters, while men accounted for 21.4%, mostly husbands. The majority of caregiving relationships were spousal (71.4%), followed by filial relationships (28.6%) (Table 1).

Regarding educational attainment (Table 1), 57.1% had completed primary school, 14.3% had completed lower secondary education, 21.4% had completed upper secondary education, and 7.1% had higher education. In terms of employment status, 57.1% were retired, 21.4% were unemployed, and 21.4% were employed (note: employment status was not differentiated further) (Table 1).

As shown in Table 2, the mean CBS (Caregiver Burden Scale) score was 72.35 (SD = 10.944), with these scores ranging from 61 to 98. The analysis revealed high levels of burden across the sample, with all 14 participants classified as experiencing intense caregiver burden.

Table 2 presents the average scores for each dimension of the CBS, including the minimum and maximum values. The average scores in all four dimensions indicate intense burden levels. The highest mean scores were found in the “expectations of caregiving” and “perceived self-efficacy” dimensions, suggesting that these aspects significantly contribute to overall burden. On a Likert scale of 1 to 5, the dimensions with the highest average scores were the impact of caregiving (3.30) and expectations of caregiving (3.98).

These findings confirm previously documented evidence of an elevated caregiver burden in Alzheimer’s caregiving contexts [19,22,23]. This sample further highlights the vulnerability of informal caregivers, reflecting the intense demands associated with dementia care and reinforcing the need for targeted support.

Regarding perceived health status (Table 3), 71.4% of the participants rated their health as “satisfactory” and 28.6% as “good”.

Following the intervention, when participants were asked about their health perception (Table 3), 71.4% reported it as “good” and 28.6% reported it as “satisfactory”—representing an inversion compared to the initial data.

A Wilcoxon signed-rank test was conducted to compare their health perceptions before and after the intervention: Z= −2.449 and *p* = 0.014, indicating a statistically significant difference. This suggests that perceived health status changed significantly following the intervention, as the burden levels decreased.

Regarding caregiver burden, a significant reduction in CBS scores was seen after the intervention. Among the 14 participants who initially had intense burden scores, 2 transitioned to no burden and 10 to a mild burden. The post-intervention mean CBS score was 50—a decrease of 28 points compared to the initial average of 78—corresponding to the classification of a “mild burden.” However, two participants remained in the “intense burden” category, despite substantial improvements (from 88 and 98 to 57 and 68, respectively). Overall, these findings indicate a meaningful reduction in caregiver burden following the intervention.

Table 4 presents the post-intervention scores by CBS dimension, including the minimum and maximum values. A reduction in the mean scores was observed across all dimensions, contributing to an overall decrease in the total burden. The most notable reductions were in the “impact of caregiving” (mean score = 2.27), “interpersonal relationships” (1.92), and “perceived self-efficacy” (1.85) dimensions.

Table 5 compares the mean scores for each CBS dimension before and after the intervention. A reduction was observed in all dimensions. The greatest decrease was seen in the “impact of caregiving” dimension, while the lowest reductions occurred in “expectations of caregiving” and “perceived self-efficacy.”

A Wilcoxon signed-rank test was also applied to comparing the CBS dimensions before and after the intervention: Z = −3.299; *p* = 0.001. This revealed a statistically significant difference. The program aimed to reduce the caregivers’ burden, and the results confirmed significant reductions across all CBS dimensions.

Notably, in the “impact of caregiving” dimension, all participants showed a decrease in scores. However, in other dimensions, such as “perceived self-efficacy,” ties were observed, meaning that for five participants, their scores remained unchanged post-intervention. Despite this, the structured intervention produced positive and statistically significant results, contributing to participant satisfaction and reinforcing its effectiveness.

In the second, qualitative phase of this explanatory sequential mixed-methods study, the participants’ reflections were analyzed to contextualize and enrich the quantitative findings further. The reflective prompts completed at the end of the intervention aimed to capture the subjective experiences and perceived impacts of the program.

Participants were asked to complete open-ended prompts such as “What I liked most about this program”, “Through this program, I discovered that…”, “At the end of this program, I feel that…”, “What I liked least was…”, and “This program helped me to…”. The most frequently identified responses included the following:

Some illustrations:

“What I liked most”: “I liked being listened to”; “It felt so good to have someone to talk to”; “I liked the techniques we practiced—I still use them”; “The words shared during the sessions were beautiful and comforting”; “I enjoyed learning to relax on my own”; “Watching the video with the children uplifted me”; “Creating the self-care kit was very meaningful.

“Through this program, I discovered that…”: “I realized I need to care for myself and do things I enjoy”; “I know myself better, I feel less bitterness and more patience in caregiving”; “I discovered positive aspects about myself—it’s good to speak about who we are”; “Saying ‘I am a caregiver’ carries a lot of power”; “Writing helps me—when I look at what I wrote, I feel relief.”

“At the end of this program, I feel that…”: “I feel very calm”; “It is so important to talk about myself and my experience”; “Every time I speak, I understand and accept myself more.”

“What I liked least”: “Honestly, nothing”; “I liked everything”; “I truly enjoyed it all.”

“This program helped me to…”: “Better manage myself and my relationship with him”; “Understand that if I am well, I can care better”; “Commit to valuing myself.”

The responses were subjected to a thematic analysis [20] to identify recurrent themes indicative of the program’s influence on the caregivers’ well-being. From the thematic analysis of these reflections, four core domains emerged as indicators of the intervention’s perceived impact:

- Recognition of the importance of narrating the caregiving experience: Caregivers acknowledged the value of expressing what it means to be a caregiver—both in terms of naming their role and narrating their personal journey;
- Increased awareness of the need for self-care: The participants expressed the importance of engaging in enjoyable and restorative activities, indicating a shift toward self-investment and resilience-building;
- Improved emotional awareness and regulation: Many reported an enhanced ability to recognize and manage their emotions, suggesting strengthened self-knowledge and internal loci of control;
- Greater patience and empathy in caregiving: Several caregivers described feeling more patient, indicating a meaningful transformation in their caregiving attitude and emotional availability.

The four informal caregivers who initially rated their health as “satisfactory” and maintained the same perception post-intervention highlighted their self-awareness of self need.

### Follow-Up Observations

One month after the intervention, follow-up contact was made with all participants. At this point, all caregivers reported maintaining self-care routines, particularly relaxation techniques and personal activities. The follow-up interaction appeared to reinforce their commitment to self-care and served as a motivational reminder of their progress.

In the two cases where the participants still reported intense burden levels, a psychological referral was suggested but declined. Nonetheless, both agreed to more frequent follow-up contact, which was implemented to provide additional support and sustain their engagement with self-care strategies.

These qualitative findings help explain the significant reductions observed in the caregiver burden scores and provide evidence of personal transformation, emotional empowerment, and behavioral changes facilitated by the Curae de Mim program.

## 4. Discussion

This study aimed to implement and evaluate a personalized psychoeducational intervention—Curae de Mim (Care for Me)—designed to reduce the caregiver burden and enhance the emotional resilience among informal caregivers of people with Alzheimer’s disease.

In line with the literature, the caregivers were predominantly female (78.6%), mainly wives or daughters, while only 21.4% were male, typically husbands. This gender distribution is consistent with prior research highlighting the feminization of informal caregiving roles in dementia contexts [19,22,23,24,25].

At the baseline, all 14 participants scored within the range of an intense burden on the CBS, confirming the findings from national and international studies on caregiving in Alzheimer’s disease [19,21,22]. This reflects the structural and emotional demands associated with long-term caregiving for individuals requiring continuous and complex support.

Following the intervention, there was a notable reduction in caregiver burden: 10 participants shifted from an intense to a mild burden, and 2 reported no burden. The mean CBS score decreased from 78 to 50, a 28-point drop, reinforcing the effectiveness of the program. Although two participants remained in the intense burden category, their scores also declined substantially (from 88 and 98 to 57 and 68, respectively), indicating significant improvements even in more severe cases.

This finding aligns with the growing body of literature supporting psychoeducational and nurse-led interventions as effective strategies for mitigating caregiver stress and enhancing mental health outcomes [24,25,26,27,28]. These improvements were not only quantitative but also corroborated by qualitative data, which provided insights into how caregivers internalized and applied what they learned during the sessions.

The thematic analysis of the reflective narratives revealed four core themes [20] that helped explain the quantitative outcomes: Recognition of caregiving as an identity and a role: The participants expressed that naming and discussing their experience as caregivers gave them a sense of validation and empowerment. Commitment to self-care: Caregivers described newfound recognition of the importance of self-care, engaging in activities that promoted emotional and physical well-being. Improved emotional regulation and awareness: Many noted greater self-understanding and emotional clarity, contributing to enhanced resilience and capacity to cope with stress. Increased patience and relational sensitivity: The caregivers reported being more tolerant and better equipped to manage caregiving dynamics compassionately. These themes align with the dimension-specific reductions observed in the CBS scores post-intervention (Table 5). The most significant changes occurred in the “impact of caregiving” domain (from 3.30 to 2.27 on the Likert scale), followed by reductions in “interpersonal relationships”, “perceived self-efficacy”, and “expectations of caregiving”. These results suggest that the intervention effectively targeted both the emotional and relational burdens associated with caregiving. These results echo evidence from recent studies that supports the impact of structured psychoeducational programs on alleviating caregiver stress [29,30].

Additionally, the follow-up contact one month after the intervention confirmed the persistence of the behavior changes: all participants maintained self-care routines such as relaxation practices and leisure activities. This sustained behavioral change suggests not only an immediate benefit but also a medium-term impact when reinforced through continued contact, echoing the findings by Taylor and Nguyen [30] on habit retention following narrative-based interventions.

The integration of narrative-based strategies—including reflective writing and group dialogue—played a critical role in facilitating emotional processing and meaning-making. These methods helped the caregivers reorganize their internal narratives, foster psychological insight, and build resilience, consistent with previous findings on the therapeutic value of narratives in high-stress caregiving contexts [30,31,32,33].

The leadership of a mental health and psychiatric nurse specialist was central to the intervention’s success. The structured, recovery-oriented group sessions enabled the caregivers not only to acquire coping tools but also to develop greater awareness of their emotional responses and relational patterns. This aligns with evidence supporting nurse-led programs that foster adaptive functioning and psychological well-being [13,33,34,35].

Moreover, the program demonstrated alignment with five of the eight Specialized Standards of Quality in Mental Health Nursing outlined by the Portuguese Order of Nurses [35]: Client satisfaction: The participants reported a strong sense of trust, support, and value in the group process. Health promotion: The program helped identify protective factors and raise awareness of emotional vulnerabilities. Prevention of complications: By addressing caregiver burden early, the intervention served as a preventive strategy aligned with evidence-based practice. Well-being and self-care: The caregivers were empowered to prioritize their health and manage their caregiving demands more autonomously. Adaptation: The program supported the development of adaptive coping strategies in the face of long-term caregiving stress.

The convergence of quantitative improvements, qualitative insights, and sustained follow-up outcomes confirms the program’s effectiveness in meeting its objectives. The findings also underscore the importance of combining structured psychoeducational content with narrative and relational approaches to maximizing the impact of caregiver support interventions.

Despite limitations related to the sample size and single-site implementation, the results are promising and support the replication of similar interventions in other primary care contexts. Future research should explore its long-term impacts, scalability, and adaptability across diverse populations.

## 5. Limitations

This study presents several limitations that must be acknowledged. Firstly, it was conducted with a small, non-probabilistic sample of 14 participants from a single community healthcare unit in central Portugal. As such, although the findings offer meaningful insights, they are not generalizable to all informal caregivers. Instead, the emphasis is on their transferability to similar contexts, as is appropriate for qualitative and mixed-methods research.

Secondly, while the explanatory sequential mixed-methods design allowed for the robust integration of quantitative and qualitative data, the absence of a control group limited the ability to attribute the observed changes solely to the intervention. The improvements noted—particularly in caregiver burden—may have been influenced by external factors, although the qualitative data strongly support the intervention’s impact.

Thirdly, the qualitative component relied on self-reported reflections and field notes, which may be subject to social desirability and recall bias. However, triangulation of the data sources (CBS scores, narratives, and observational notes) and the use of reflexive journaling by the researcher strengthened the validity of the interpretations.

Fourth, the follow-up period was limited to one month, which did not allow for conclusions about the sustainability of the changes in caregiver burden or self-care behaviors over time. While the follow-up feedback indicated continued use of the strategies learned, longitudinal data are needed to assess its long-term impact.

Finally, the design and implementation of the program were shaped by the cultural, organizational, and professional standards of the Portuguese healthcare system, including the role of the mental health and psychiatric nurse specialist. These factors may influence the feasibility and applicability of the intervention in other healthcare systems or cultural contexts.

Future studies should aim to replicate this intervention in varied settings with larger, more diverse samples; consider experimental or quasi-experimental designs; and incorporate extended follow-up periods to evaluate the durability and scalability of the outcomes.

## 6. Conclusions

The aim of this study was to implement and evaluate Curae de Mim (Care for Me), a personalized psychoeducational intervention designed to reduce the caregiver burden and strengthen the emotional resilience in informal caregivers of individuals with Alzheimer’s disease. Delivered through a series of individualized sessions, the intervention was structured around core psychoeducational themes, while being tailored to each caregiver’s emotional needs, caregiving context, and reflective insights.

The findings demonstrate that the intervention achieved its objective: the quantitative analysis showed a statistically significant reduction in the caregivers’ burden, with a mean ESC score reduction of 28 points, and the qualitative data revealed transformative shifts in their emotional regulation, self-awareness, and self-care practices. The Wilcoxon signed-rank test confirmed the significance of the changes observed across the CBS dimensions, particularly in the impact of caregiving and interpersonal relationship domains.

Through the integration of narrative reflection, the caregivers articulated their experiences, reconstructed meaning, and gained insight into their caregiving identity. The thematic analysis identified consistent themes—recognition of emotional needs, value of self-care, improved emotional management, and increased relational patience—which directly explained and reinforced the quantitative improvements. These outcomes demonstrate the unique value of narrative-based, person-centered mental health nursing in bridging the gap between structured content and lived experience.

The program aligns closely with five of the eight Specialized Quality Standards in Mental Health Nursing defined by the Portuguese Order of Nurses—specifically in promoting client satisfaction, health promotion, complication prevention, well-being, and psychosocial adaptation. The individualized nature of the intervention allowed for flexibility and responsiveness, reinforcing the principles of humanized, personalized care in community-based nursing practice. These standards were evidenced through participant feedback, the sustained behavioral changes at the follow-up, and their improved emotional resilience.

While this was a small-scale, single-site study, its methodological transparency, use of explanatory sequential mixed methods, and detailed outcome reporting offer strong preliminary support for its replication. The follow-up conducted one-month post-intervention provided additional confirmation of sustained self-care behaviors and perceived benefit, reinforcing the program’s practical relevance.

Importantly, the intervention was personalized, with each caregiver receiving individual attention and guidance from a mental health nurse specialist. This format enabled the therapeutic strategies to be tailored to unique caregiving challenges and psychological profiles. The Curae de Mim (Care for Me) program offers a replicable and scalable model of holistic, evidence-based nursing intervention tailored to the complex psychosocial realities of dementia caregiving. It underscores the critical role of mental health and psychiatric nurses in primary care settings and advocates for the integration of personalized, recovery-oriented strategies to support family caregivers.

Future research should build on these results by extending the duration of the follow-up, diversifying the settings and populations, and exploring integration with wider mental health service pathways. It should be ensured that personalized support for carers becomes a standard component of dementia care. Also, future research should focus on the longitudinal outcomes, multi-site implementation, and adaptation to diverse sociocultural caregiving contexts, thereby strengthening the evidence base and practical applicability of this innovative approach to community mental health support.

## Figures and Tables

**Table 1:** Demographic characteristics of informal caregivers.

Variable	Mean	SD	Min	Max
Age (years)	61.5	11.72	45	80
Years as caregiver	3.64	3.52	1	11
Daily caregiving hours	18.21	6.7	5	24
	N (%)			
Sex				
Female	11 (78.6)			
Male	3 (21.4)			
Relationship to care recipient				
Spouse	10 (71.4)			
Child	4 (28.6)			
Education level				
Primary Education	8 (57.1)			
Upper Secondary	2.0 (14.3)			
High School	3 (21.4)			
Higher Education	1 (7.1)			
Professional status				
Retired	8 (57.1)			
Employed	3 (21.4)			
Unemployed	3 (21.4)			

**Table 2:** Dimension scores of the CBS.

CBS Dimensions	Mean Score	SD	Max	Min	Mean (Likert 1–5)
Impact of caregiving (items 1–3, 9–13, 17–18, 22)	36.35	6.27	48	29	3.30
Interpersonal relationships (items 4–6, 16, 19)	14.28	3.96	22	9	2.85
Expectations of caregiving (items 7–8, 14–15)	15.92	2.97	20	10	3.98
Perceived self-efficacy (items 20–21)	5.70	2.22	10	2	2.89

**Table 3:** CBS scores.

Health Perception	Before—n (%)	After—n (%)
Unsatisfactory	0 (0.0)	0 (0.0)
Satisfactory	10 (71.4)	4 (28.6)
Good	4 (28.6)	10 (71.4)
Very Good	0 (0.0)	0 (0.0)

**Table 4:** CBS dimension scores after the intervention.

CBS Dimensions	Mean Score	SD	Max	Min	Mean (Likert 1–5)
Impact of caregiving (items 1–3, 9–13, 17–18, 22)	25.07	4.23	34	19	2.27
Interpersonal relationships (items 4–6, 16, 19)	9.64	1.82	14	7	1.92
Expectations of caregiving (items 7–8, 14–15)	12.28	2.94	18	7	3.07
Perceived self-efficacy (items 20–21)	3.71	1.13	6	2	1.85

**Table 5:** Comparison of CBS dimension scores before and after intervention.

CBS Dimensions	Mean Score (Before)	Mean Score (After)
Impact of caregiving (items 1–3, 9–13, 17–18, 22)	36.35	25.07
Interpersonal relationships (items 4–6, 16, 19)	14.28	9.64
Expectations of caregiving (items 7–8, 14–15)	15.92	12.28
Perceived self-efficacy (items 20–21)	5.70	3.71

## Data Availability

The original contributions presented in this study are included in the article. Further inquiries can be directed to the corresponding author.

## References

[1] World Health Organization (2017). Global Strategy and Action Plan on Ageing and Health.

[2] Organisation for Economic Co-Operation and Development (2011). Help Wanted? Providing and Paying for Long-Term Care.

[3] Brodaty H., Donkin M. (2009). Family caregivers of people with dementia. Dialogues Clin. Neurosci..

[4] Schulz R., Sherwood P.R. (2008). Physical and mental health effects of family caregiving. Am. J. Nurs..

[5] Fonareva I., Oken B.S. (2014). Physiological and functional consequences of caregiving for relatives with dementia. Int. Psychogeriatr..

[6] Lethin C., Renom-Guiteras A., Zwakhalen S., Soto-Maritin M., Saks K., Zabalegui A., Challis D.J., Nilsson C., Karlsson S. (2017). Psychological well-being over time among informal caregivers caring for persons with dementia living at home. Aging Ment. Health.

[7] Sequeira C., Sampaio F., Sequeira C. (2020). Intervenção psicoeducativa com cuidadores informais. Cuidar de Pessoas com Demência: Um Guia Para Cuidadores Informais.

[8] Government of Canada (2019). A Dementia Strategy for Canada: Together We Aspire.

[9] Department of Health (2009). Living Well with Dementia: A National Dementia Strategy.

[10] Federal Ministry for Family Affairs, Senior Citizens, Women and Youth (2021). Pflegezeitgesetz (Care Leave Act).

[11] Australian Government (2022). Carer Gateway: Support for Carers.

[12] Direção-Geral da Saúde (2020). Programa Nacional para a Saúde Mental 2020.

[13] Silaule O., Casteleijn D., Adams F., Nkosi N.G. (2024). Strategies to Alleviate the Burden Experienced by Informal Caregivers of Persons with Severe Mental Disorders in Low- and Middle-Income Countries: Scoping Review. Interact. J. Med. Res..

[14] Barker P., Buchanan-Barker P. (2005). The Tidal Model: A Guide for Mental Health Professionals.

[15] Tschudin V. (2013). Ethics in Nursing: The Caring Relationship.

[16] George T. Mixed Methods Research: Definition, Guide & Examples. https://www.scribbr.com/methodology/mixed-methods-research/.

[17] Barker P. (2001). The tidal model: Developing an empowering, person-centered approach to recovery within psychiatric and mental health nursing. J. Psychiatr. Ment. Health Nurs..

[18] Andresen R., Oades L., Caputi P. (2003). The experience of recovery from schizophrenia: Towards an empirically validated stage model. Aust. N. Z. J. Psychiatry.

[19] Sequeira C.A. (2007). Adaptação e validação da Escala de Sobrecarga do Cuidador para a população portuguesa. Rev. Ref..

[20] Braun V., Clarke V. (2006). Using thematic analysis in psychology. Qual. Res. Psychol..

[21] Lincoln Y.S., Guba E.G. (1985). Naturalistic Inquiry.

[22] Gonçalves-Pereira M., Zarit S.H. (2014). The Zarit Burden Interview in Portugal: Validity and recommendations in dementia and palliative care. Acta Médica Port..

[23] Gómez-Gallego M., Gómez-Gallego J.C. (2021). Predictors of caregiver burden of patients with Alzheimer disease attending day-care centres. Int. J. Environ. Res. Public Health.

[24] Rodríguez-Madrid M.N., del Río-Lozano M., Fernández-Peña R., García-Calvente M.d.M. (2021). Changes in caregiver personal support networks: Gender differences and effects on health (CUIDAR-SE study). Int. J. Environ. Res. Public Health.

[25] Kim A., Woo K. (2022). Gender differences in the relationship between informal caregiving and subjective health: the mediating role of health promoting behaviors. BMC Public Health.

[26] Frias C.E., Garcia-Pascual M., Montoro M., Ribas N., Risco E., Zabalegui A. (2020). Effectiveness of a psychoeducational intervention for caregivers of people with dementia with regard to burden, anxiety and depression: A systematic review. J. Adv. Nurs..

[27] Del-Pino-Casado R., López-Martínez C., Orgeta V. (2019). Sense of coherence, burden and mental health in caregiving: A systematic review and meta-analysis. J. Affect. Disord..

[28] Sánchez-Martínez V., Cauli O., Corchón S. (2024). Long-term caregiving impact and self-care strategies in family caregivers of people with neuropsychiatric disorders: A mixed-method study. Diseases.

[29] Cintoli S., Tommasini L.L., Del Prete E., Pignataro S., Cerratti F., Petti M.C. (2024). The psychoeducational interventions: A valuable communication tool to support the caregiver of people with dementia. BMC Geriatr..

[30] Ma Y., Gong J., Zeng L., Wang Q., Yao X., Li H., Chen Y., Liu F., Zhang M., Ren H. (2023). The Effectiveness of a Community Nurse-Led Support Program for Dementia Caregivers in Chinese Communities: The Chongqing Ageing and Dementia Study. J. Alzheimer’s Dis. Rep..

[31] Ando M., Kukihara H., Yamamoto M., Ninosaka Y., Saito N. (2019). Development of narrative approach for family caregiver’s QOL at home hospice and contents of narrative. Psychology.

[32] Cheng S.T., Li K.K., Losada A., Zhang F., Au A., Thompson L.W., Gallagher-Thompson D. (2020). The effectiveness of nonpharmacological interventions for informal dementia caregivers: An updated systematic review and meta-analysis. Psychol. Aging.

[33] McAdams D.P., McLean K.C. (2013). Narrative Identity. Curr. Dir. Psychol. Sci..

[34] Haigh C., Hardy P. (2011). Tell me a story—A conceptual exploration of storytelling in healthcare education. Nurse Educ. Today.

[35] Ordem dos Enfermeiros (2015). Regulamento n.º 356/2015 de 25 de Junho. Regulamento dos Padrões de Qualidade dos Cuidados Especializados em Enfermagem de Saúde Mental.

